# Good mental health for people with intellectual disabilities: a participatory focus group study

**DOI:** 10.1186/s12939-025-02562-8

**Published:** 2025-06-18

**Authors:** Sophie Komenda-Schned, Sarah Jasmin Landskron, Paula Moritz, Nicole Braunstein, Josef Hochmeister, Karin Riegler, Robert Saugspier, Louise Hillenkamp, Brigitte Lueger-Schuster, Luis Salvador-Carulla, Elisabeth Lucia Zeilinger

**Affiliations:** 1 Department of Clinical and Health Psychology, Faculty of Psychology, University of Vienna, Liebiggasse 5, Vienna, A-1010 Austria; 2 Vienna Doctoral School in Cognition, Behavior and Neuroscience, University of Vienna, Vienna, Austria; 3 Research Institute for Developmental Medicine, Johannes Kepler University Linz, Linz, Austria; 4 Bundesverband der Lebenshilfe Österreich, Vienna, Austria; 5 Health Research Institute, Faculty of Health, University of Canberra, ACT, Canberra, Australia; 6 Department of Clinical Research SBG, Academy for Ageing Research, Haus der Barmherzigkeit, Vienna, Austria

**Keywords:** Intellectual disabilities, Good mental health, Focus groups, Participatory research, Thematic analysis, Co-researchers, Experts by experience, Self-advocates

## Abstract

**Background:**

Mental health is a fundamental component of overall health. However, it remains unclear whether commonly accepted definitions of good mental health, like the WHO´s, are applicable to people with intellectual disabilities (ID). While there are studies with experts in the field of mental health and ID investigating this issue, the perspectives of people with ID themselves have yet to be adequately represented in this discourse.

**Aim:**

We aimed to identify key factors contributing to good mental health in people with ID, based on the perspectives of people with ID as experts on their own account.

**Methods:**

Five focus groups with people with ID (*N* = 20) from different living and working settings were conducted. The data was analyzed and synthesized using reflexive thematic analysis. Following a participatory research approach, people with ID acted as co-researchers throughout the research process, including data analysis.

**Results:**

We found six themes constituting good mental health in people with ID: (1) physical health, (2) working and living environment, (3) social contacts, (4) appropriate support, (5) competencies, and (6) psychosocial functioning. Psychosocial functioning included five sub-themes: (6a) emotions, (6b) self-determination and self-concept, (6c) doing something meaningful, (6d) responding well in social situations, and (6e) having energy and being able to relax.

**Conclusions:**

The findings widely align with the factors outlined in the WHO definition of good mental health. While working productively as well as contributing to the community were not discussed, the relevance of social contacts and individualized support was emphasized. Additionally, this study underscores the added value of considering the unique perspectives of people with ID as experts on their own account and as co-researchers in participatory research settings.

**Supplementary Information:**

The online version contains supplementary material available at 10.1186/s12939-025-02562-8.

## Accessible summary

• In research on health and disabilities, the views of people with learning disabilities* are often overlooked. We wanted to know: What is good mental health for people with learning disabilities?

• We carried out 5 group discussions on good mental health. 20 people with learning disabilities participated in the group discussions. Together with our co-researchers with learning disabilities, we summarized the discussions.

• What is important for good mental health?

1. Keeping the body healthy.

2. Having a good working and living environment.

3. Feeling connected and having close social contacts.

4. Getting the support you need.

5. Having competencies and learning new things.

6. Psychosocial aspects (e.g., having energy and being able to relax,

7. doing something meaningful, responding well in social situations)

• Our participants with learning disabilities said. that feeling close to other people is very important. They also said that getting the right kind and amount of support. is necessary to stay mentally healthy.

• Working with co-researchers is good for everybody: Academic researchers learn to consider other perspectives. Co-researchers gain insights into disability research and are empowered to speak up.

* The term learning disabilities is widely used by people with intellectual disabilities to refer to their own group.

* We use the words “learning disabilities” instead of intellectual disabilities, because that is how people with intellectual disabilities call themselves. When we say learning disabilities we mean intellectual disabilities.

## Introduction

People with intellectual disabilities (ID) are characterized by reduced intellectual functioning (IQ < 70) and adaptive behavior. Four levels of ID can be distinguished (mild, moderate, severe, and profound), depending on intellectual abilities and adaptive behavior. Moreover, ID arise before the age of 18 years [1, 2].

For the general population there are numerous definitions of good mental health, with the most common one being the World Health Organization’s, which defines good mental health as “a state of wellbeing in which every individual realizes his or her own potential, can cope with the normal stresses of life, can work productively and fruitfully, and is able to make a contribution to her or his community” [3]. Due to their disability, people with ID experience limitations in functioning domains, like adaptive skills, health, or participation [4–8]. Despite their intellectual and behavioral limitations, people with ID can experience good mental health. Yet, there are no specific definitions or conceptualizations of good mental health in this population [9, 10], and it remains unclear whether mental health definitions for the general population are suitable for an ID population as well.

People with ID often lack opportunities to shape their (social) environment. They are likely to depend on support in some areas of their life, including their working environment, forms of assisted living, mobility, or other activities of daily living [4, 11, 12], and their choice of roommates or support persons, which represent a large part of their social network [13], is limited. A mismatch between a person’s needs and his/her environmental demands acts as one of multiple risk factors for the development of psychiatric disorders in people with ID [14]. Other risk factors include stigmatization, increased rates of abuse or traumatic experiences, limited social skills and coping strategies [15–17]. Prevalence rates of psychiatric disorders are up to two to seven times higher than in people without ID [18, 19], yet specialized prevention and intervention strategies for this population are rare. While there have been some efforts regarding the treatment of psychiatric disorders and challenging behavior in this population [15, 20, 21], the lack of evidence-based theories on good mental health for people with ID still hinders the development of appropriate mental health preventive and intervention strategies and policies.

Previous research suggests that environmental as well as psychosocial factors are excellent starting points for evidence-based strategies to foster good mental health in people with ID [10–12, 22]. A recent systematic literature review showed that absence of mental illness, physical health, environmental factors, and psychosocial functioning are constituting factors of good mental health in this population [10, 12]. An expert interview study [11, 12] supported these findings [10] and provided deeper insights. In particular, it emphasized the significance of consistent and reliable social relationships, especially in the context of support services. Moreover, the importance of providing individualized, needs-oriented support as well as opportunities for personal growth were stressed by the experts [11, 12].

However, with data from scientific literature and mental health experts alone, definite conclusions about determining factors of good mental health of people with ID cannot be drawn, because these prior studies did not include the perspectives of people with ID. Triangulating data by asking several groups of participants is considered to encourage richness and depth of the data and thereby allowing for a more comprehensive understanding of the construct in question [23, 24]. This has proven to be especially useful in exploratory, qualitative research in an ID population [22]. Moreover, people with mild to moderate ID have been shown to provide reliable responses to mental health questions, when being supported adequately [e.g., adapted rating scales, easy-to-read language;,^25^].

The primary aim of this study was to explore the key factors that contribute to good mental health in an ID population, based on the perspectives of people with ID as experts on their own account. The study was designed as a participatory project, with people with ID as co-researchers.

## Methods

### Participants

In five separate focus groups 20 people with ID participated (*n* = 3–5 per group). The distribution of participants within the focus groups is depicted in Table 1. Their age ranged from 23 to 82 years (*Mean =* 51.3, *SD =* 16.57). 55% of the participants identified as male. Most participants lived in a supported living facility (55%) or in their own apartment with at least some professional support (30%), the others resided with their family (5%), their partner (5%) or in their own apartment without professional support (5%). Due to their age 25% of the participants were unoccupied (retired), 70% worked in a sheltered workshop and one person (5%) had a protected workplace.

**Table 1:** Allocation of participants within the focus groups

focus group number	participant numbers
1	participant 1–4
2	participant 5–7
3	participant 8–12
4	participant 13–17
5	participant 18–20

### Measures

#### Suitability of focus groups for participants with ID

Focus groups capture the perspectives of groups that are socially marginalized or underrepresented in the general population by addressing exploratory questions in under-researched areas [26, 27]. Compared with other more structured data collection methods, focus groups are closer to natural conversations [27] and are easier to access for individuals with ID or other marginalized populations [28]. The social interactions within focus groups encourage participants to exchange experiences and express opinions that might not surface in individual interviews [29–31]. Moreover, discussing their experiences can empower participants [32, 33].

#### Focus group guidelines

Semi-structured guidelines for the focus groups were developed by trained researchers in the field of mental health and intellectual disability (SKS, PM). Questions were open-ended, allowing participants to express their views freely. They addressed the following topics: (1) definition of psyche / soul, (2) definition of good mental health, (3) promoting good mental health (see Supplementary Files 1 and 2).

#### Focus group materials and accessibility

Each focus group included two hypothetical case studies of Klara and Konrad, developed with our co-researchers, illustrating examples of the everyday life of people with differing levels of abilities. This approach enables the participants to identify differences in the definition and promotion of mental health between different ranges of functioning. In addition, participants were given a printed table of all the topics and METACOM pictograms [version 8;,^34^] mentioned during the discussions. Following nominal group technique [28, 35], participants were asked to rate the importance of each topic. The focus groups guidelines, the case studies, and the voting sheet are provided in the Supplementary Files 1 and 2.

### Procedure and sampling strategy

Support organizations for people with ID with varying support needs and self-advocate groups in Vienna were contacted. E-mails with attached invitation letters and flyers in easy-to-read language were sent to organizations and self-advocates. The organizations distributed the flyers, and potential participants or their caregivers contacted the researchers directly.

A total of five focus groups were conducted in July and August 2023. After providing written informed consent, participants were asked to complete demographic information presented in easy-to-read language. Focus groups were conducted by three trained members of the research team, including (clinical) psychologists with experience with people with ID (SKS, SJL, PM). Audio recordings were made during each session, and notes were taken by a team member. One member of the research team documented participants’ responses in real time using keywords and images. These visual summaries were presented via a projector during the focus groups. TRINT [36] was used to aid transcription. Focus groups lasted between 39 and 66 min, with an average duration of 47 min. Two focus groups were conducted in university rooms, three were held at the participant’s facilities. The study was approved by the Ethics Committee of the University of Vienna (No. 00885).

### Data analysis

Quantitative, sociodemographic data was analyzed using SPSS [v. 29.0.1.0;,^37^]. Qualitative interview data was analyzed using thematic analysis (Braun and Clarke [38],. The coding followed an inductive approach and was carried out by three researchers (LH, SKS, SJL). MAXQDA [v. 22; 39] was used to facilitate analysis. We utilized a relativistic, experimental, and semantic approach, focusing exclusively on the participants’ perspectives, guided by the data itself rather than by theoretical assumptions. For focus groups one and two, initial codes were developed collaboratively, and the coding tree was constructed inductively. Focus groups three to five were coded individually by the three researchers, discrepancies were thereafter resolved in a group discussion. Subsequently, all focus groups were reviewed, novel codes were applied across all datasets, and uncertainties were resolved through team consensus. The final codes were translated into easy-to-read language for the co-researchers. The co-researchers assisted with clustering and finalizing the themes in a collaborative process. Therefore, a puzzle analogy was applied: the translated codes were written on puzzle pieces and clustered in a joint session of the (co-)researchers (NB, LH, JH, SKS, SJL, PM, RS, KR). Since the final themes were named together with the co-researchers, they are written in easy-to-read language even though some of them resemble psychological constructs (e.g., social competence is named responding well in social situations).

### Participatory research including co-researchers with ID

**Fig. 1:**
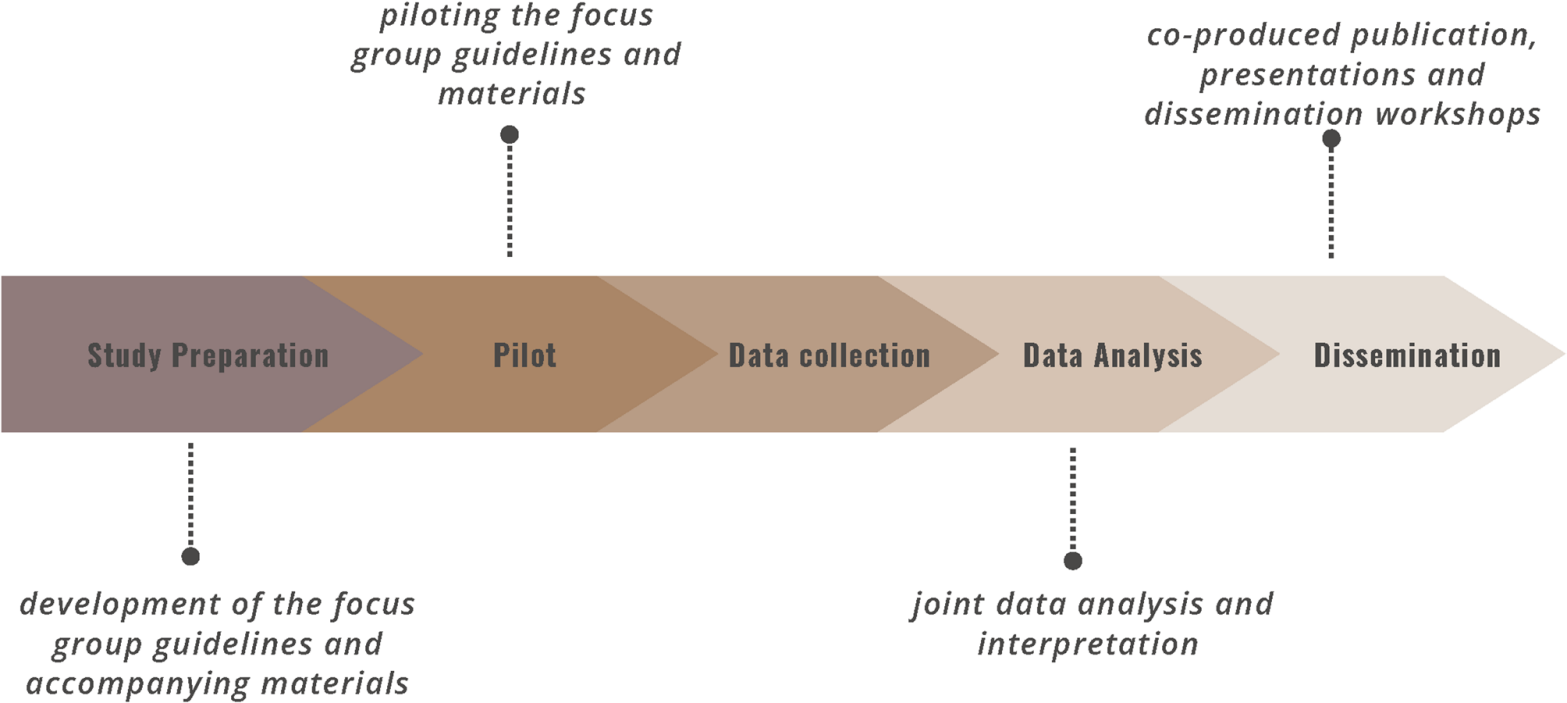
Overview of the participatory research process

Four co-researchers with ID (NB, JH, KR, RS) were involved in (1) the development of the focus group guidelines and accompanying materials, (2) the piloting of the focus group guidelines, (3) the data analysis, and (4) the publication of the study’s results. Regular team meetings were held online or in person approximately every four to eight weeks. In a respectful working environment, communication took place at eye level and all (co-)researchers’ competencies could come into play. The co-researchers’ life experiences and the other authors’ academic knowledge are seen as equally valuable for the research process [40–44]. An overview of the participatory process is presented in Fig. 1.

Participatory research settings are beneficial for researchers and co-researchers likewise: Considering the unique perspectives of people with ID and their living reality, allows for higher chances to address and to understand the needs of the target population, which in turn impacts the motivation to participate in a study as well as the acceptance of the study’s results within an ID community [44, 45]. Moreover, easily accessible materials and methods facilitate the conduction of a study with people with ID. For co-researchers with ID, involvement in participatory research can have an empowering effect [41, 44], following the main principle of the people first movement “nothing about us, without us” [46].

## Results

**Fig. 2:**
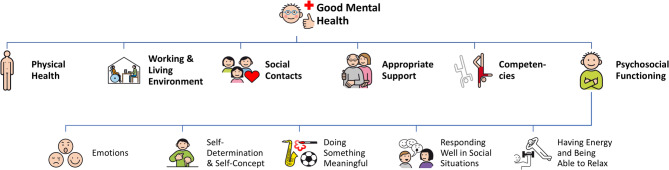
Themes and sub-themes of good mental health

Six themes constituting good mental health in people with ID were derived from the data: (1) physical health, (2) working and living environment, (3) social contacts, (4) appropriate support, (5) competencies, and (6) psychosocial functioning. Psychosocial functioning was the richest theme, it was therefore split up into five sub-themes: (6a) emotions, (6b) self-determination and self-concept, (6c) doing something meaningful, (6d) responding well in social situations, and (6e) having energy and being able to relax. The coding tree is depicted in Fig. 2.

Additionally, one side-theme was identified: confusing mental health with mental illness. When first asked about their associations with good mental health, many participants’ responses revolved around aspects of mental illness. They talked about different mental disorders and their symptoms. Moreover, various treatment options were discussed (e.g., taking medication, seeking the help of a psychologist/psychotherapist, visiting or calling counseling services). One participant stated that.

*“Mental health is*,* for example*,* when I have serious problems*,* that I go to a psychologist and talk about all my problems*,* […] like what’s bothering me or weighing me down*,* and maybe*,* I don’t know*,* get some advice or tips*,* like what I can do about it and so on.”* (participant 8).

### Physical health

Different facets of physical health such as sleep, nutrition and doing exercises are crucial for mental health according to the participants. *“Sleeping well is healthy”* (participant 4). Having good sleeping-habits, including the choice of when to go to bed and when to wake up, are important aspects for recovery and being in a positive mood. However, disturbed sleep can be due to unresolved issues. One participant described: “*yes*,* when something is really bothering me […] […] it keeps going around in my head forever*,* and then I can’t fall asleep […] then I’m not feeling well again.”* (participant 8). The participants reported that healthy eating habits and a balanced diet with vitamins and fibers are equally important for mental health. Enjoying food and drinks is not only a source of physical nourishment but also provides moments of enjoyment and relaxation, contributing positively to mental health. Engaging in sports can promote both physical and mental health as stated by the participants: “Moderator: *Would you say that sports also help with your wellbeing? That it’s good for your mental health as well? Or does sports only help the body?* Participant 2: *It helps me feel good.”* Also, moderate activities, like walking, offer valuable mental health benefits such as calming the mind and organizing thoughts. Regular preventive medical check-ups and the use of necessary medication enable long-term mental health and a sense of feeling good.

### Working and living environment

Having and getting a job, whether in a sheltered workshop or another setting, and a positive working environment, including good relationships with colleagues, promotes mental health through aspects such as earning money and social connections. Participants reported on specific strategies for responding to needs and emotions during work to directly communicate and maintain health: *“we have a mood barometer at work*,* where we are supposed to write down how we’re feeling each day”* (participant 10). One participant stated that *“if you feel bad*,* you can talk to us [caregivers] anytime”* (participant 11).

How individuals feel about their living environment has a significant impact on their mental health. One participant expressed satisfaction with the situation:

*“We’re happy with our [supported living] apartment. We’re doing well. […] I enjoy it. I don’t have any problems.”* This participant also emphasized the value of home: “*home is where it’s the nicest*” (participant 2).

The need for personal space, privacy, and the ability to withdraw when necessary are essential for maintaining mental health. One participant shared their frustration regarding a lack of privacy, stating, “participant 9: *„my sister walks in every time […] and is always listening when I’m talking to someone on the phone.”* This illustrates how constant interruptions can lead to a feeling of discomfort and personal space being invaded. Another participant pointed out a strategy for dealing with noise: *“If it’s too loud*,* I go to my room.“* (participant 15).

### Social contacts

Participants emphasized the importance of social interactions and the impact of others on one’s mental health, with one participant suggesting that others should *“not make fun of him [Konrad*,* the person in the case study]*,* [and] encourage him”* (participant 6) so that he can believe in his abilities and not feel like a failure. Community integration through shared spaces in assisted living facilities helps to counteract loneliness, joint leisure activities foster inter-connection. Being connected to others encompasses various forms of support, including visiting services for elderly people, colleagues at work, and positive relationships with professional caregivers. These connections involve being understood and having the ability to talk to others. Engaging in conversations about problems with support persons and participating in group leisure activities, further enhances these connections and contributes to one’s mental health.

The aspect of being close to others delves deeper into the quality and intimacy of relationships. This involves cultivating strong friendships and family ties that provide emotional support. The ability to discuss personal matters openly with friends contributes positively to mental health. Additionally, love and romantic relationships provide an essential element in promoting emotional proximity and mutual affection, with one participant noting: *“Love is also part of health for me.”* (participant 15).

Family relations reinforce emotional support through regular visits, shared activities and physical closeness. Many participants emphasized the importance of hugs for emotional comfort: *“What’s also important*,* is hugs to make me feel better. […] From my sisters*,* my nieces and nephews. From my aunt. Basically*,* from the whole family.”* (participant 7). By offering company and opportunities for physical activity, such as walking the dog or horse riding, also pets contribute to emotional wellbeing, providing comfort and security.

### Appropriate support

Appropriate support encompasses emotional, practical, and social assistance tailored to individual needs. Emotional support means receiving reassurance and “*comforting each other when someone is feeling down.*” (participant 6). Meaningful examples are *“when she [caregiver] notices I’m not doing well*,* she hugs me*,* and that really helps me.”* (participant 10). Practical support involves assistance with daily tasks like *“[…] helping out around the house. Taking care of the animals […] taking out the trash and changing the bed linens […]”* (participant 2). Another participant stated, *“I always get a bath at the weekend.”* (participant 15) and linked this to feeling good. Participants indicated training to enhance independence, as well as the provision of assisted and alternative forms of communication as further facets of practical support. Moreover, financial support from family members (e.g., to pay for therapy sessions) was valued by the participants. Giving and receiving support (e.g., from friends, family, caregivers) contributes to a sense of mental health. Participants emphasized that they are *“helping each other”* (participant 7), another one shared: *“I enjoy supporting people […] so they can have a sense of wellbeing and mental health.”* (participant 3).

Asking for support is crucial to being mentally healthy: *“Get support*,* have a conversation. You need that to stay healthy too.”* (participant 12). While it is essential to *“talk to your caregivers and ask them to help you”* (participant 11), it can be embarrassing or difficult. However, one participant expressed the importance: *“I have to dare*,* yes*,* I need the support*,* because if he never gets support because he doesn’t dare*,* then he will always fall by the wayside.”* (participant 12).

Moreover, appropriate support was the only topic where the participants figured that there are differences between our fictional cases Konrad, with low functioning levels, and Klara, with high functioning levels. While the participants noted that Konrad and Klara share the same needs regarding their mental health, they concluded that Konrad and Klara have to be provided with different levels of individualized, needs-oriented support, in order to do so.

### Competencies

To learn basic competencies such as reading, writing and calculating enhances mental health according to the participants: “Participant 15: *I can read incredibly well*,* I spent hours reading at my parents’ place.* Moderator: *Is that also something that makes you feel good? Reading?* Participant 15: *Yes.*” Furthermore, skills of mobility, using public transport, getting around on your own were mentioned. Competencies also include being able to do activities of daily living independently (e.g., house keeping, cleaning, grocery shopping). One participant expressed pleasure: *“What I enjoy is cooking a lot and always cleaning the kitchen”* (participant 10).

Establishing routines and waking up early for work, is a way to feel comfortable; for example, one participant noted, *“I always get up at 5 o’clock. That’s the norm for me”* (participant 18). Learning about health, including first aid and knowing how to call emergency services, empowers individuals and contributes to mental health.

### Psychosocial functioning

Psychosocial functioning was the richest theme with five sub-themes.

#### Emotions

Emotional awareness and regulation play a central role in psychosocial functioning for mental health, as participants emphasized in mood, feelings, and coping strategies. It’s not just about feeling consistently happy, participants also noted experiencing a range of emotions (e.g., anger, sadness, joy) and expressing them: “Participant 19: *Crying. Crying.* Moderator: *Crying? Mmh. Crying also has something to do with the psyche?* Participant 19: *Yes (…) Also laughing.* Moderator: *Laughing too?* Participant 19: *Yes.*”

Strategies to deal with emotions and feelings were also mentioned as a part of mental health, for instance: *“I like watching animal videos when I’m angry.”* (participant 11). However, difficulties with emotion regulation are also apparent: *“Usually*,* when I’m angry or grumpy or something like that. I can’t completely control it*,* and then I might accidentally snap. Even if I don’t want to sometimes.”* (participant 8). Participants also described dealing with loss as a component of emotion regulation: *“My dad passed away before my mom. So*,* we were always at the cemetery. In the beginning*,* I would cry whenever I went there.”* (participant 14). It was discussed that grieving became easier over time, when corresponding emotions are processed rather than suppressed.

#### Self-determination and self-concept

Self-determination and self-concept are relevant for participants’ sense of identity and mental health, encompassing aspects such as autonomy, independence, self-worth and confidence, and standing up for oneself. The participants emphasized the importance of independence, such as getting around using public transportation (see 3.5. competencies). The importance of freedom of choice was stressed, regarding decisions in daily life but also in the medical field, for example the choice of therapy. As one participant noted: *“[I want to] decide everything for myself”* (participant 5). Self-worth and self-confidence were discussed in the context of trying new things, like finding out what someone is capable of which in turn strengthened the self-confidence and a sense of self-worth. “Moderator: *You said that she should try out what she can do? Why would that be good for her mental health?* Participant 6: *So that she feels more worthy. And self-confidence. That’s important*.”

Another aspect is the ability to stand up for oneself, including talking about inequalities and communicating directly with others who do harm. One participant explained: *“If something bothers you*,* you can speak up. Tell people. You don’t have to bottle up your feelings all the time.”* (participant 11). Participants expressed the importance of setting personal boundaries, as exemplified *“Yes*,* and when [first name] annoys me*,* I say*,* “Go away*,* leave me alone*,* I don’t want this.”* (participant 9), as well as to defend oneself, and *“I told him that I actually feel threatened by him because I don’t know what he wants.”* (participant 11).

#### Doing something meaningful

Engaging in meaningful activities that provoke a sense of purpose and pride represent important facets of good mental health for the participants, whether in leisure or work settings (e.g., being a group-representative at your sheltered workplace). For instance, a participant shared: *“Like [name of organization] I’m also a representative there*,* the deputy chairman. These are the important things where I say*,* oh*,* I have to work on that now.”* (participant 12). Another one described value in setting personal goals: *“I’m taking part in the [marathon] again next year. That’s important to me.”* (participant 20).

Focusing on joyful leisure activities are characterized as beneficial. A participant reported the worth of fresh air and going on vacation with friends, family, or colleagues from work to describe the importance of being outside or in nature: *“The air was so good for me there [on vacation]. I didn’t need a headache medicine that often”* (participant 15). Participants figured that it helps to see a different environment, to be somewhere else, and not think about work. Trying out new activities was highly valued by people with ID, not only for its enjoyment as an influence on mental health but also in the context of fostering self-confidence and self-worth (see 3.6.2. self-determination and self-concept). Lastly, attending church was mentioned in a group of elderly participants as a meaningful activity.

#### Responding well in social situations

The ability to effectively solve problems and handle conflicts with others represents a part of social competence. Skills like deescalating conflicts, seeking the help of professionals (e.g., psychologists), expressing emotions and needs in case of conflicts were highlighted by the participants. Getting along with others was essential: *“[…] understanding each other well. Getting along well with each other. And harmonize well”* (participant 2). Empathy and understanding in considering the needs of others as well as taking the perspective of others contribute to mental health. However, it is equally important that others are considerate of people with ID and their learning difficulties (e.g., give them more time to complete a task) - *“it’s important to respond to people’s needs and don’t just say ‘no*,* you have to do this because I told you so’”* (participant 10).

#### Having energy and being able to relax

The participants also considered having energy and being able to relax to be highly decisive for mental health: *“When I’m feeling good? Then I can tear down anything in my way.”* (participant 12), and *“[…] when I feel good*,* I want to do lots of things*,* I’m full of energy. I laugh a lot*,* make jokes*,* and am just in a good mood [laughing]”* (participant 8). Having fun and laughing were mentioned as another aspect of mental health: *“laughing a lot is healthy”* (participant 19). Relaxation and avoiding stress were equally crucial. The importance of calmness and tranquility were emphasized: *“for example*,* at the [swimming pool] or wherever*,* you can just lie down on the grass and simply enjoy the day”* (participant 8) and the negative aspects of stress highlighted: *“Stress is deadly*,* as far as I know”* (participant 18). Other participants valued taking time for oneself to take a break and to calm down by explaining: *”When I’m not mentally or (…) overwhelmed*,* then I take some time for myself.”* (participant 12). Likewise, it was discussed that motivation is essential, both through encouragement from others and because of personal interests. Being motivated by somebody to do (challenging) things is described as needed in some situations: *“Yes*,* when someone motivates me to go to work*,* go to the doctor*,* or tackle challenges. […] Because sometimes I need that kind of motivation. So that I actually do something*,* you know?”* (participant 3). On the other hand, aspects of intrinsic motivation such as having an interesting job or doing something that is close to one’s heart promote mental health as well as motivating others.

### Prioritizing the most important aspects of good mental health

When asked for the most important factors for good mental health, the focus group participants concurringly prioritized (1) social contacts, especially regarding being connected to others, (2) doing something meaningful, (3) having energy and being able to relax, and (4) physical health.

## Discussion

This study provides initial insights into the perspectives of people with ID as experts on their own account, identifying key factors contributing to good mental health in this population using a participatory research approach. The results align broadly with the WHO definition of good mental health [3]. Mental health as a state of wellbeing is reflected in the subthemes such as having energy and being able to relax, emotions (e.g., experiencing positive emotions), and doing something meaningful (e.g., joyful activities, vacations). Realizing one’s own potential aligns with (sub-)themes such as competencies, self-determination and self-concept (e.g., learning to do things independently), and doing something meaningful (e.g., trying out new things). Coping with stress is reflected in appropriate support (e.g., asking for support), emotions (e.g., emotion regulation), and responding well in social situations (e.g., solving interpersonal conflicts).

**Fig. 3:**
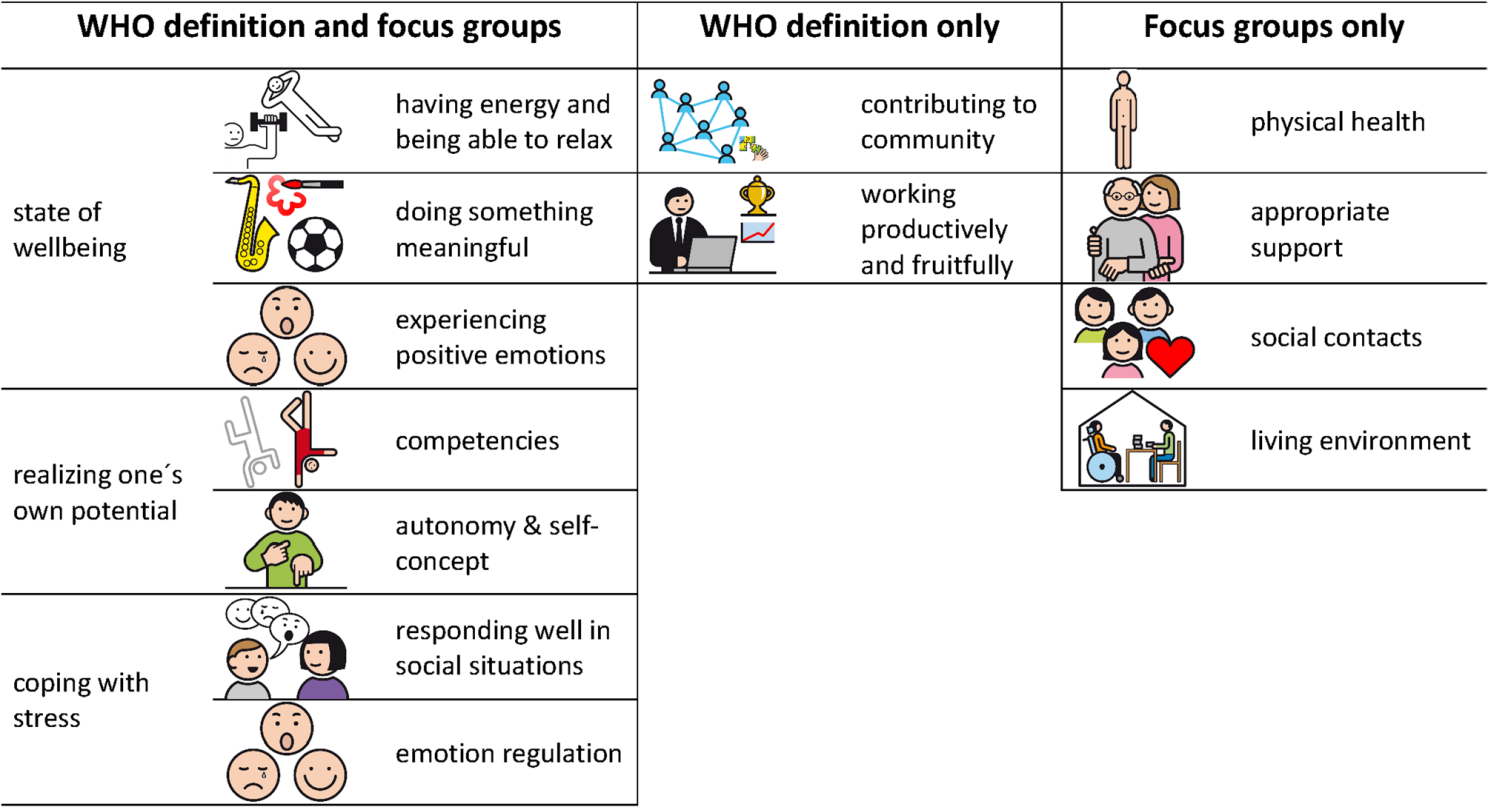
Comparison of the study’s results and the WHO’s definition of good mental health

However, aspects of the WHO definition such as contributing to the community and working “productively and fruitfully” were not explicitly discussed. While participants valued a positive working environment, their focus was on *having a job*, enjoying it, and maintaining *positive relationships* with colleagues and support persons. This aligns with research on relatedness and job satisfaction in people with ID [47]. Aspects like job performance or output were not brought up by the participants. These elements might take on a different meaning due to the contextual differences in the lives of people with ID. It presumably reflects limited choices and opportunities for people with ID, particularly in sheltered workshops where providing a sense of stability and social interaction take priority over more traditional expectations of productivity, which was also captured in our data. Regarding community, connections with others were highlighted. Group activities or simply being with others were proposed to prevent loneliness and foster a sense of connection or feeling included, contributing positively to mental health. While congruent with most of the WHO’s definition, some novel aspects of good mental health for people with ID were identified, such as physical health, appropriate support and social contacts. Two of these themes were even decided to be among the most relevant ones. The perspectives of our participants may have been shaped by politically and socially desirable discourse and demands for a differentiated understanding taking into account the specific circumstances and needs of people with ID in western societies. Moreover, as opposed to people without ID, people with ID may experience more challenges in navigating daily life and tend to depend more on support services and specialized programs. In this context, political and societal aspects might also have contributed to the findings of this study. For a detailed comparison of the study’s results with the WHO’s definition see Fig. 3.

Social contacts, irrespective of the setting (work, living, leisure) were described as one of the most important aspects of good mental health for people with ID. This mirrors prior findings on the importance of relationships with roommates and support persons [13, 48–50]. Corroborating existing studies [51, 52], participants also emphasized balancing *privacy* and *connection* within their living environments, indirectly addressing the need for a suitable living space. A further prioritized aspect of good mental health was the engagement in meaningful activities, including leisure and job-related activities. In line with previous research, this highlights the positive effects of meaningful activities on mental health [11, 53, 54].

The subtheme having energy and being able to relax was a central aspect of good mental health within the focus groups and the co-researchers. The co-researchers suggested naming it “stands above everything else”, highlighting its relevance. Subsequently, we focused on its primary content and collaboratively agreed on naming it “having energy and being able to relax”. In contrast to the topic’s high relevance for people with ID, this aspect was not mentioned in a prior study with mental health experts [11]. This discrepancy may arise because this theme describes the experience of mental health (e.g., positive affect), while other themes represent prerequisites for it. To a large extent, this subtheme corresponds to the construct of hedonic wellbeing (i.e., describing positive affect and it’s behavioral correlates), which alongside with eudaimonic wellbeing (i.e., positive functioning and meaningfulness in life) represents a traditional approach to good mental health [22, 55–57].

Autonomy, self-determination, and independence were also identified as essential themes. Participants used autonomy and self-determination interchangeably, though not in the sense of basic needs theory [58, 59]. Nevertheless, their emphasis on autonomy aligns with research showing its positive association with wellbeing [48, 58, 59].

For people with ID opportunities for self-determination, autonomy, and independence oftentimes depend on support persons and/or settings [4, 48]. Our participants indicated that varying levels of ID—and thus levels of functioning [6, 8]—are not linked to differences in personal needs to maintain and promote good mental health. They rather determine their support needs, to realize healthy activities and behaviors. In line with previous research, our participants stressed the importance of providing needs-oriented, person-centered support for people with ID [43, 60–62].

The side-theme of confusing *mental health* with *mental illness* reflects a common societal misconception and can also be observed in empirical studies [30, 63–67]. Mental health experts also reported this confusion [11], but differences emerged: Experts emphasized environmental and structural factors, while people with ID focused on individual experiences, such as *competencies*, *job satisfaction*, *self-advocacy*, and *having energy*. These contrasting perspectives highlight the need to integrate diverse viewpoints for a comprehensive understanding of good mental health in people with ID.

### Limitations and strengths

While the relatively large sample size and minimal changes to the coding tree after analyzing the first two focus groups indicate code saturation, the results might vary with different samples. The study primarily focused on individuals with mild to moderate ID, excluding those with severe disabilities, who would require alternative methods to participate such as individual interviews or augmented and alternative communication. Even within this study, verbal abilities varied among the participants resulting in an unequal distribution of contribution, favoring more skilled speakers.

Nevertheless, the study’s balanced age and gender distribution enhances rigor. Importantly, it is the first study on good mental health to directly consult people with ID using a participatory research framework. Collaborative sessions with co-researchers, employing creative and accessible methods to lower the threshold for participation, represent an innovative approach to qualitative research. It not only strengthens the study’s credibility and relevance by incorporating the perspectives and experiences of people with ID but also fosters a sense of empowerment among the co-researchers through their active involvement in the research process. We encourage other researchers to engage in participatory research that includes adaptive and reflective methodological approaches to extract these benefits.

### Future research

Future research should aim to integrate the divergent perspectives of people with ID, as well as those of other key stakeholders such as caregivers or support professionals. Triangulating these viewpoints can provide a more comprehensive understanding of good mental health, with consensus-building as a potential research focus.

## Conclusions

People with ID consider specific aspects as relevant for their good mental health. Social contacts, learning basic competencies, and appropriate, needs-oriented support, as well as having energy and being able to relax are particularly important for people with ID. Taking into account different views, especially those of the target group themselves as experts on their own account, leads to differentiated results which could contribute to the development of specific health promotion programs for people with ID that truly meet the mental health needs of this population. This approach is not only expected to improve health outcomes and to reduce mental disorders and challenging behavior [e.g., 68], but also aligns with the UN Convention of Rights of Persons with Disabilities [UN CRPD, 69], advocating for the full inclusion and mental health of people with ID. Furthermore, it underscores the value of a participatory research approach to actively incorporate the perspectives of people with ID, encouraging inclusion, and ensuring their opinions and lived experiences remain central to the research process.

## Electronic supplementary material

Below is the link to the electronic supplementary material.

Supplementary Material 1

Supplementary Material 2

## Data Availability

Due to the nature of the research and to protect the privacy and anonymity of participants, data cannot be shared.
