# Supplementary material for: Good mental health for people with intellectual disabilities: a participatory focus group study
Source: Int J Equity Health. 2025 Jun 18;24:180. doi: 10.1186/s12939-025-02562-8 (PMC12175403; doi:10.1186/s12939-025-02562-8)
Supplement: Supplementary file 1 — Supplementary Material 1 [file 12939_2025_2562_MOESM1_ESM.pdf]

Supplementary File: Focus Group Materials (English Version)

Attachments

Focus Group Guidelines 2
Case Study Klara 6
Case Study Konrad 7
Voting Sheet 8
Example of a Completed Voting Sheet 9

Focus Group Guidelines

- In advance: completing the socio-demographic questionnaire

Welcome

- Welcome & introduction of the team
- Temporal overview of group discussion
- Clarification of the setting (procedure, breaks, recording, traffic light cards, leaving the room, using the first name/last name)
- Reference to informed consent

Introductions & Wellbeing Round → *Dixit-Cards*

- Introduction & wellbeing round: Name, how am I feeling right now?

Thematic Introduction → *live Metacom- Documentation*

- Start recording!

What is health?

Explanation Health:
There are many opinions on this,
what health is.
Some people say,
health is the opposite of illness.
Others say:
As long as a person is alive,
a person always has healthy and sick parts within oneself.
You are never just sick or just healthy

Mental Health → *live Metacom-Documentation*

What does mental/soul mean? or What is the psyche/soul?

What is mental health?

How can you tell that you are mentally healthy?

What helps you to be/stay mentally healthy/ How can you improve your mental health?

What do you need from others to stay mentally healthy?

BREAK 5‘

Case Study Klara: high-functioning → *live Metacom-Documentation*

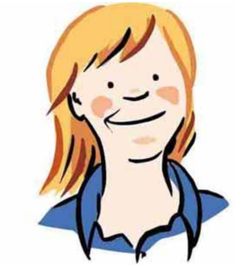

Klara is a 32-year-old woman with learning disabilities.
She is living in her own apartment.
Her assistant comes to visit her twice a week
and helps her with the housework.
Klara can do a lot on her own,
she needs little help in everyday life.

She works in a supermarket.
Klara takes the tram to and from work every day.
She practiced this before with her assistant.

She is healthy and feels well.
She makes sure that she stays physically healthy.
She goes for a walk every day,
and sometimes to play soccer.
She often eats healthy snacks like fruit or vegetables.
She also makes sure that he stays mentally healthy.

What do you think Klara is doing for it?

**Is there anything else Klara needs
to stay mentally healthy?**

**Which kind of support does Klara need from others
to stay mentally healthy?**

Case Study Konrad: low-functioning → *live Metacom-Documentation*

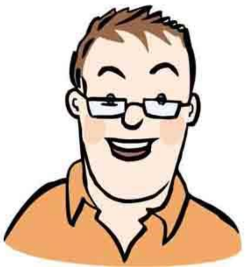

Konrad is a 32-year-old man with learning disabilities.
He lives in a accommodation facility
for people with learning disabilities.
Konrad needs a lot of support in his everyday life.
His caregivers are there for him day and night.

Konrad works in a sheltered workshop.
The transport service picks him up every day at 9 a.m.
and brings him back in the afternoon every day.

Konrad can not read.
He has a weekly schedule with pictures.
This allows him to see
what he will be doing each day.

On Monday, Konrad likes to go for a walk with a caregiver.
On Thursday, he goes shopping together with his caregivers and roommates.
There are often healthy snacks in the accommodation facility
like fruit or vegetables.

Konrad is healthy and feels well.
He makes sure that she stays physically healthy.
He also makes sure that he stays mentally healthy.

What do you think Konrad is doing for it?

Is there anything else Konrad needs to stay mentally healthy?

Which kind of support does Konrad need from others to stay mentally healthy?

BREAK 15'

**Voting**	→ *traffic light cards, Metacom-Doc.*

- Introduction: we are interested in what you consider to be the most important aspects of mental health; there is no right/wrong answer

- Step 1: Having all factors assessed
- Step 2: Top 3

green = is very important to me
yellow = is a bit important to me
red = is not important to me

**Closing**

- Brief summary of the topics
- Emphasizing the positive

How are you feeling now? (offer debriefing if necessary)

Is there anything else you would like to tell us?

- Thank you & goodbye

Sociodemographics: Personal Information

I am

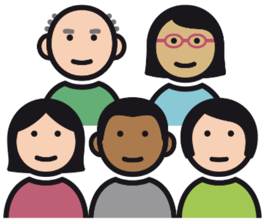

- ☐ a woman
- ☐ a man
- ☐ diverse
- ☐ I do not want to say that.

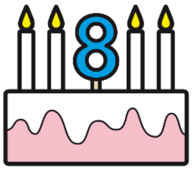

I am ____________________________ years old.

I live …

- ☐ in a supported living facility.
- ☐ with my family.
- ☐ with my partner.
- ☐ in my own apartment with professional support.
- ☐ in my own apartment without professional support.
- ☐ others: _____________________

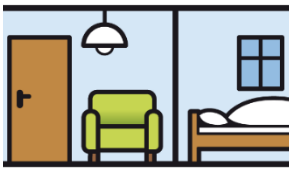

I work …

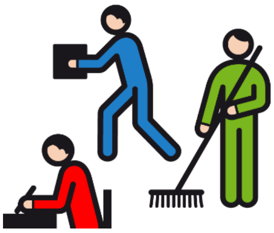

- ☐ in a sheltered workshop.
- ☐ in a protected workplace.
- ☐ In the open job market.
- ☐ I do not work.
- ☐ others: _____________________

Case Study Klara

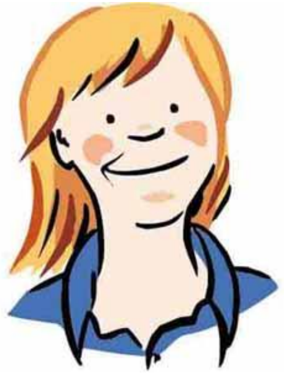

Klara is a 32-year-old woman with learning disabilities.
She is living in her own apartment.
Her assistant comes to visit her twice a week
and helps her with the housework.
Klara can do a lot on her own,
she needs little help in everyday life.

She works in a supermarket.
Klara takes the tram to and from work every day.
She practiced this before with her assistant.

She is healthy and feels well.
She makes sure that she stays physically healthy.
She goes for a walk every day,
and sometimes to play soccer.
She often eats healthy snacks like fruit or vegetables.
She also makes sure that he stays mentally healthy.

Questions:

What do you think Klara is doing for it?

Is there anything else Klara needs
to stay mentally healthy?

Which kind of support does Klara need from others
to stay mentally healthy?

Case Study Konrad

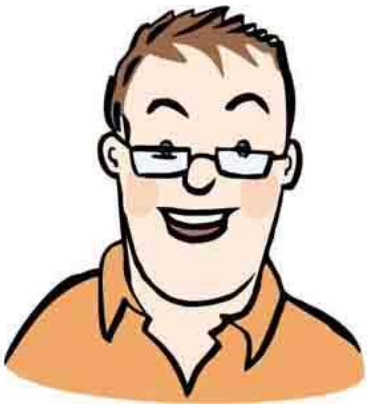

Konrad is a 32-year-old man with learning disabilities.
He lives in a accommodation facility
for people with learning disabilities.
Konrad needs a lot of support in his everyday life.
His caregivers are there for him day and night.

Konrad works in a sheltered workshop.
The transport service picks him up every day at 9 a.m.
and brings him back in the afternoon every day.

Konrad can not read.
He has a weekly schedule with pictures.
This allows him to see
what he will be doing each day.

On Monday, Konrad likes to go for a walk with a caregiver.
On Thursday, he goes shopping together with his caregivers and roommates.
There are often healthy snacks in the accommodation facility
like fruit or vegetables.

Konrad is healthy and feels well.
He makes sure that she stays physically healthy.
He also makes sure that he stays mentally healthy.

Questions:

What do you think Konrad is doing for it?

Is there anything else Konrad needs
to stay mentally healthy?

Which kind of support does Konrad need from others
to stay mentally healthy?

Voting Sheet

Picture	Description	1st Vote	2nd Vote
		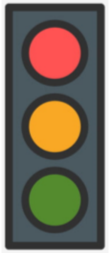	
		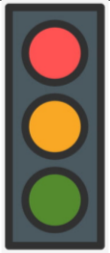	
		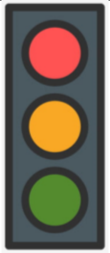	
		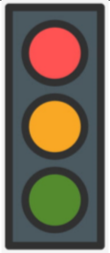	

Example of a Completed Voting Sheet

Picture	Description	1st Vote	2nd Vote
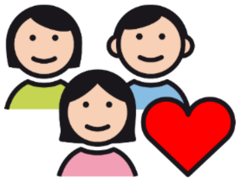	Family and friends	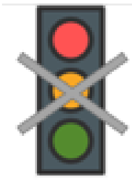	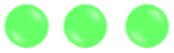
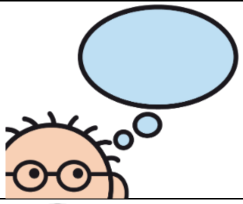	Thinking positive	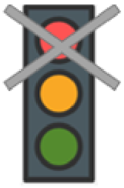	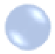
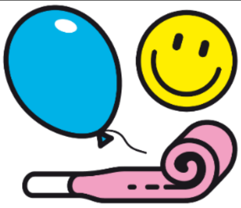	Having Fun	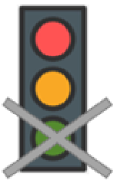	
