# Supplementary material for: Good mental health for people with intellectual disabilities: a participatory focus group study
Source: Int J Equity Health. 2025 Jun 18;24:180. doi: 10.1186/s12939-025-02562-8 (PMC12175403; doi:10.1186/s12939-025-02562-8)
Supplement: Supplementary file 2 — Supplementary Material 2 [file 12939_2025_2562_MOESM2_ESM.pdf]

Supplementary File: Focus Group Materials (German Version)

Anlagen

Fokusgruppe Leitfaden 2
Soziodemografie: Angaben zur Person 5
Fallbeispiel Klara 6
Fallbeispiel Konrad 7
Abstimmung 8
Beispiel ausgefüllter Abstimmungsbogen 9

Fokusgruppe Leitfaden

- vorab: Fragebogen Soziodemografie ausfüllen lassen

Begrüßung

- Begrüßung & Vorstellung des Teams
- zeitl. Überblick über Gruppendiskussion
- Aufklärung über Setting (Ablauf, Pausen, Aufnahme, Ampelkarten, Raum verlassen, Duzen/Siezen)
- Hinweis auf Informed Consent

Vorstellungs- & Befindlichkeitsrunde → *Dixit-Karten*

- Vorstellungs- & Befindlichkeitsrunde: Name, wie geht es mir gerade?

Thematischer Einstieg → *live Metacom-Dokumentation*

- Start Aufnahme!

Was ist Gesundheit?

Erklärung Gesundheit:
Es gibt viele Meinungen dazu,
was Gesundheit ist.
Manche Menschen sagen,
Gesundheit ist das Gegenteil von Krankheit.
Andere sagen:
Solange ein Mensch lebt,
hat er immer gesunde und kranke Teile in sich.
Man ist nie nur krank oder nur gesund

Psychische Gesundheit → *live Metacom-Dokumentation*

Was bedeutet psychisch/seelisch? bzw. Was ist die Psyche/Seele?

Was ist psychische Gesundheit?

Woran merken Sie, dass du psychisch gesund bist?

Was hilft Ihnen dabei, psychisch gesund zu sein/zu bleiben?/ Wie können Sie Ihre psychische Gesundheit verbessern?

Was brauchen Sie von anderen, um psychisch gesund zu bleiben?

PAUSE 5‘

Fallbeispiel Klara: high-functioning → *live Metacom-Dokumentation*

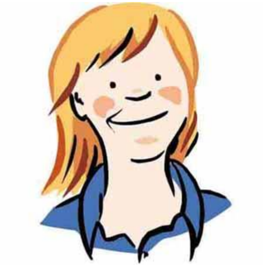

Klara ist eine 32-jährige Frau mit Lern-Schwierigkeiten.
Sie lebt in ihrer eignen Wohnung.
2x pro Woche kommt ihre Unterstützerin zu Besuch
und hilft ihr mit dem Haushalt.
Klara kann vieles alleine, sie braucht wenig Hilfe im Alltag.

Sie arbeitet in einem Supermarkt.
Klara fährt jeden Tag alleine mit der Straßenbahn in die Arbeit und zurück.
Das hat sie vorher mit ihrer Unterstützerin geübt.

Sie ist gesund und ihr geht es gut.
Sie achtet darauf, dass sie körperlich gesund bleibt.
Sie geht jeden Tag eine Runde spazieren
und manchmal zum Fußball spielen.
Sie isst oft gesunde Sachen wie Obst und Gemüse.

Klara schaut auch darauf, dass sie psychisch gesund bleibt.

Was denken Sie, macht Klara dafür?

**Gibt es noch etwas, das Klara braucht,
um psychisch gesund zu bleiben?**

**Welche Unterstützung braucht Klara von anderen,
um psychisch gesund zu bleiben?**

Fallbeispiel Konrad: low-functioning → *live Metacom-Dokumentation*

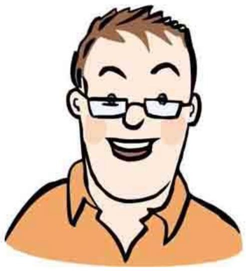

Konrad ist ein 32-jähriger Mann mit Lern-Schwierigkeiten.
Er lebt in einem Wohnhaus für Menschen mit Lern-Schwierigkeiten.
Konrad braucht viel Hilfe im Alltag.
Seine Betreuer sind Tag und Nacht für ihn da.

Konrad arbeitet in einer Werk-Statt.
Der Fahrten-Dienst holt ihn jeden Tag um 9 Uhr ab
und bringt ihn nachmittags zurück.

Konrad kann nicht lesen.
Er hat einen Wochenplan mit Bildern.
So kann er nachschauen, was er jeden Tag machen wird.

Am Montag geht Konrad gerne mit einem Betreuer spazieren.
Am Donnerstag geht er mit Betreuern und Mitbewohnern gemeinsam einkaufen.
Im Wohnhaus gibt es oft gesunde Sachen, wie Obst und Gemüse.

Konrad ist gesund und ihm geht es gut.
Er achtet darauf, dass er körperlich gesund bleibt.
Konrad schaut auch darauf, dass er psychisch gesund bleibt.

Was denken Sie, macht Konrad dafür?

**Gibt es noch etwas, das Konrad braucht,
um psychisch gesund zu bleiben?**

**Welche Unterstützung braucht Konrad von anderen,
um psychisch gesund zu bleiben?**

PAUSE 15‘

Abstimmung → *Ampelkarten, Metacom-Doku*

- Hinführung: uns interessiert, was für dich die wichtigsten Punkte zu psychischer Gesundheit sind; es gibt kein richtig/falsch

- 1. Schritt: alle Faktoren einschätzen lassen
- 2. Schritt: Top 3

grün = ist mir sehr wichtig
gelb = ist mir ein bisschen wichtig
rot = ist mir nicht wichtig

Abschluss

- kurze Zusammenfassung der Themen
- Betonung des Positiven

Wie geht es Ihnen jetzt? (ggf. Nachbesprechung anbieten)

Gibt es noch etwas, was Sie uns sagen wollen?

- Bedanken & Verabschieden

Soziodemografie: Angaben zur Person

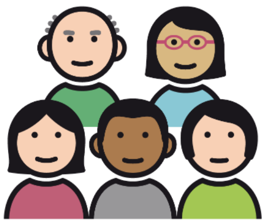

Ich bin …

- ☐ eine Frau
- ☐ ein Mann
- ☐ divers
- ☐ möchte ich nicht sagen

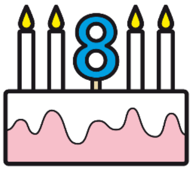

Ich bin ____________________________ Jahre alt.

Ich wohne …

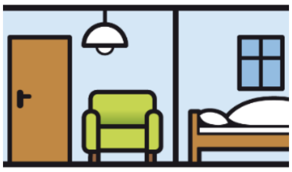

- ☐ in einem Wohn-Haus
- ☐ bei meiner Familie
- ☐ mit meiner Partnerin/mit meinem Partner
- ☐ in einer eigenen Wohnung mit Unterstützung
- ☐ in einer eigenen Wohnung ohne Unterstützung
- ☐ anders: ____________________

Ich arbeite …

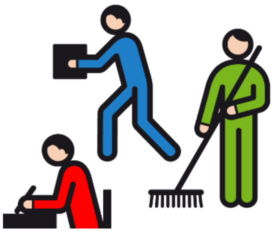

- ☐ in einer Werk-Statt
- ☐ an einem geschützten Arbeits-Platz
- ☐ am 1. Arbeits-Markt
- ☐ nicht
- ☐ anders: ____________________

Fallbeispiel Klara

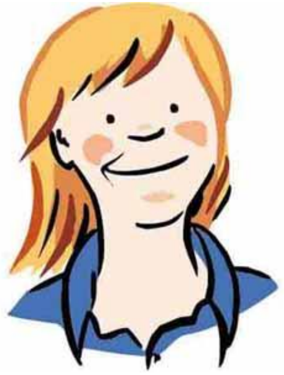

Klara ist eine 32-jährige Frau mit Lern-Schwierigkeiten.
Sie lebt in ihrer eignen Wohnung.
2x pro Woche kommt ihre Unterstützerin zu Besuch
und hilft ihr mit dem Haushalt.
Klara kann vieles alleine, sie braucht wenig Hilfe im Alltag.

Sie arbeitet in einem Supermarkt.
Klara fährt jeden Tag alleine mit der Straßenbahn in die Arbeit und zurück.
Das hat sie vorher mit ihrer Unterstützerin geübt.

Sie ist gesund und ihr geht es gut.
Sie achtet darauf, dass sie körperlich gesund bleibt.
Sie geht jeden Tag eine Runde spazieren
und manchmal zum Fußball spielen.
Sie isst oft gesunde Sachen wie Obst und Gemüse.

Klara schaut auch darauf, dass sie psychisch gesund bleibt.

Was denken Sie, macht Klara dafür?

Gibt es noch etwas, das Klara braucht,
um psychisch gesund zu bleiben?

Welche Unterstützung braucht Klara von anderen,
um psychisch gesund zu bleiben?

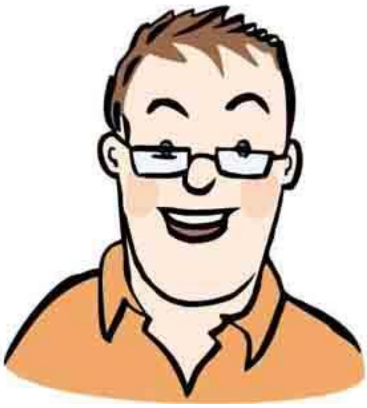

Fallbeispiel Konrad

Konrad ist ein 32-jähriger Mann mit Lern-Schwierigkeiten.
Er lebt in einem Wohnhaus für Menschen mit Lern-Schwierigkeiten.
Konrad braucht viel Hilfe im Alltag.
Seine Betreuer sind Tag und Nacht für ihn da.

Konrad arbeitet in einer Werk-Statt.
Der Fahrten-Dienst holt ihn jeden Tag um 9 Uhr ab
und bringt ihn nachmittags zurück.

Konrad kann nicht lesen.
Er hat einen Wochenplan mit Bildern.
So kann er nachschauen, was er jeden Tag machen wird.

Am Montag geht Konrad gerne mit einem Betreuer spazieren.
Am Donnerstag geht er mit Betreuern und Mitbewohnern gemeinsam einkaufen.
Im Wohnhaus gibt es oft gesunde Sachen, wie Obst und Gemüse.

Konrad ist gesund und ihm geht es gut.
Er achtet darauf, dass er körperlich gesund bleibt.
Konrad schaut auch darauf, dass er psychisch gesund bleibt.

Was denken Sie, macht Konrad dafür?

Gibt es noch etwas, das Konrad braucht,
um psychisch gesund zu bleiben?

Welche Unterstützung braucht Konrad von anderen,
um psychisch gesund zu bleiben?

Abstimmung

Piktogramm	Beschreibung	1. Abstimmung	2. Abstimmung
		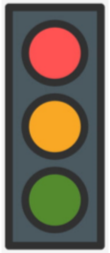	
		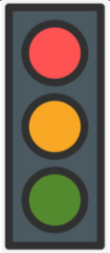	
		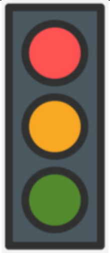	
		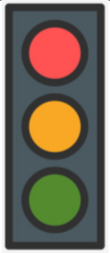	

Beispiel ausgefüllter Abstimmungsbogen

Piktogramm	Beschreibung	1. Abstimmung	2. Abstimmung
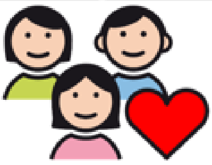	Familie und Freunde	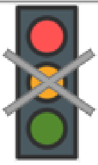	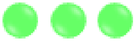
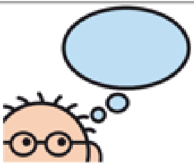	Positiv Denken	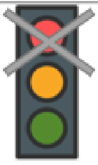	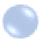
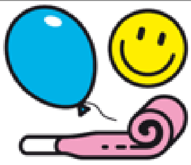	Spaß haben	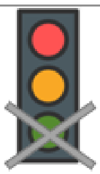	
